# Caregiver burden in patients with behavioural variant frontotemporal dementia and non-fluent variant and semantic variant primary progressive aphasia

**DOI:** 10.1007/s00702-021-02378-0

**Published:** 2021-07-19

**Authors:** Michael Guger, Stefan Raschbacher, Lukas Kellermair, Milan R. Vosko, Christian Eggers, Thomas Forstner, Karin Leitner, Alexandra Fuchs, Franz Fellner, Gerhard Ransmayr

**Affiliations:** 1 Department of Neurology 2, Med Campus III, Kepler University Hospital GmbH, Krankenhausstr. 9, 4021 Linz, Austria; 2 Medical Faculty, Johannes Kepler University, Linz, Austria; 3 Department of Applied Systems Research and Statistics, Johannes Kepler University, Linz, Austria; 4 Clinical and Health Psychology Unit, Med Campus III, Kepler University Hospital GmbH, Linz, Austria; 5 Central Radiology Institute, Med Campus III, Kepler University Hospital GmbH, Linz, Austria

**Keywords:** Behavioural variant frontotemporal dementia, Non-fluent variant primary progressive aphasia, Semantic variant primary progressive aphasia, Caregiver burden, Instrumental activities of daily living, Neuropsychiatric symptoms

## Abstract

Studies on caregiver burden in patients with frontotemporal lobar degeneration are rare, differ methodologically and show variable results. Single center longitudinal pilot study on caregiver burden and potential risk factors in patients with behavioural variant frontotemporal dementia (bvFTD) and semantic (svPPA) and non-fluent variants (nfvPPA) primary progressive aphasia. Forty-six bvFTD, nine svPPA, and six nfvPPA patients and caring relatives were analysed for up to 2 years using the Mini-Mental State Examination as global measure for cognitive performance, Frontal Assessment Battery (frontal lobe functions), Frontal Behavioural Inventory (personality and behaviour), Neuropsychiatric Inventory (dementia-related neuropsychiatric symptoms), Barthel Index and Lawton IADL Scale (basic and instrumental activities of daily living), the Caregiver Strain Index (CSI), and in most participants also the Zarit Burden Interview (ZBI). CSI baseline sum scores were highest in bvFTD (mean ± SD 5.5 ± 3.4, median 5, IQR 6), intermediate in svPPA (2.9 ± 2.3; 3; 3.5) and low in nfvPPA (1.6 ± 2.1; 1; 2). Similar differences of caregiver burden were found using the ZBI. During follow-up, CSI and ZBI sum scores deteriorated in svPPA, not in bvFTD and nfvPPA, and correlated significantly with personality and behaviour, neuropsychiatric symptoms, caregiver age, and instrumental, but not basic activities of daily living, Mini-Mental State Examination scores or frontal lobe functions. This study reveals differences in caregiver burden in variants of frontotemporal lobar degeneration. Caregivers should be systematically asked for caregiver burden from the time of the diagnosis to provide comprehensive support in time.

## Introduction

Frontotemporal lobar degeneration (FTLD) comprises a spectrum of neurodegenerative disorders characterized by atrophy of the frontal and temporal lobes and the striatum and changes in behaviour, personality, language skills, cognition and motor functions. Several clinical and neuropathological subtypes of FTLD are differentiated (Rabinovici and Miller 2010). The behavioural variant frontotemporal dementia (bvFTD) is characterized by early behavioural impairment, personality changes and a frontal-dysexecutive syndrome progressing to dementia (Rascovsky et al. 2011). Specific medical treatment options are lacking. BvFTD patients progressively lose social skills and daily living competence and require an increasing degree of support and care. Primary progressive aphasia (PPA) is a clinical syndrome with heterogeneous neuropathologic causes. The key diagnostic criterium of PPA is isolated prominent difficulties with language at symptom onset and for the initial phases of the disease (Gorno-Tempini et al. 2011). Semantic variant primary progressive aphasia (svPPA) typically presents with progressive decline of confrontation naming, single-word comprehension, object knowledge, surface dyslexia and dysgraphia and behavioural deterioration including compulsion and loss of empathy (Gorno-Tempini et al. 2011). Non-fluent variant primary progressive aphasia (nfvPPA) is characterized by loss of fluency, agrammatism, errors in speech sounds and distortions and difficulties in understanding syntactically complex sentences (Gorno-Tempini et al. 2011). Patients with svPPA may develop loss of social and emotional sensitivity and alertness early, whereas patients with nfvPPA may preserve their personality until advanced phases of the disease (Toller et al. 2018).

In most countries, the care for dementia patients is mainly provided by female family members (Schneider et al. 1999; Ransmayr et al. 2018; Besser and Galvin 2019). Professional care is usually claimed only in advanced disease phases. Some 20 years ago, a multi-national European study reported major differences between countries in the burden of care of spouses of Alzheimer’s disease (AD) patients, probably due to differences in traditions, and professional personal, medical and financial support of family care. Austrian caregivers reported severe caregiver burden, which might have been due to lack of professional and financial support of caregivers (Schneider et al. 1999). Only few studies assessed caregiver burden and underlying factors in rare dementias, in particular in persons providing care for a family member over a longer period of time (Riedijk et al. 2008; Karnatz et al. 2019). Caregiving interferes with personal needs and duties including family life, occupation, privacy, social activities and leisure and may result in significant health problems (Karnatz et al. 2019; Besser and Galvin 2019). Neuropsychiatric symptoms and behavioural deterioration contribute significantly to caregiver strain in mild to moderate AD (Schneider et al. 1999; Ransmayr et al. 2018). Neuropsychiatric and behavioural symptoms mostly differ in bvFTD and AD. Prevailing symptoms in bvFTD are early disinhibition, apathy, loss of sympathy and empathy. Similar behavioural changes may also occur in svPPA and in advanced phases of nfvPPA (Hsieh et al. 2016; Liu et al. 2017, 2018; Karnatz et al. 2019; Besser and Galvin 2019). The question arises whether impairments of behaviour and personality and neuropsychiatric symptoms contribute to caregiver burden also in bvFTD, svPPA and nfvPPA.

Comprehensive longitudinal studies on caregiver burden and underlying factors in bvFTD, svPPA and nfvPPA are rare (Riedijk et al. 2008; Hsieh et al. 2016; Liu et al. 2017; Karnatz et al. 2019). A longitudinal pilot study may contribute to a better knowledge of caregiver burden in these disorders, contribute to better medical and public awareness of rare dementias (Wynn and Carpenter 2020) and allow for larger multi-centric studies. We, therefore, performed a longitudinal study on caregiver burden and underlying factors in persons caring for family members suffering from bvFTD, svPPA and nfvPPA.

Our hypotheses were:

1. Burden of care is more severe in bvFTD than in svPPA and nfvPPA patients.
2. Caregiver burden develops differently over time in these disorders.
3. Neuropsychiatric and behavioural symptoms in bvFTD, svPPA and nfvPPA, although usually different from those in AD, are likewise prevailing factors of caregiver burden.

## Patients and methods

The study was conducted according to the Declaration of Helsinki and approved by the local ethical review board (Ethikkommission des Landes Oberösterreich; FTLA Study, Protocol No. 254). Written informed consent was obtained from the patients and their caring family members.

### Data collection

Between 2007 and 2018 consecutive patients with bvFTD, svPPA, nfvPPA, progressive supranuclear palsy, corticobasal syndrome and frontotemporal dementia in association with amyotrophic lateral sclerosis (spectrum of FTLD disorders) as well as AD and Parkinson’s disease patients, who had been referred to the neurocognitive and movement disorder clinics of the Departments of Neurology 2 of the Kepler University Hospital and the Hospital of the Mercy Friars Linz, closely cooperating primary care centers for these diagnoses, and their carers were asked for inclusion in a longitudinal registry study (FTLA study).

Until 2011, the criteria of Neary et al. (1998) were applied for the diagnosis of FTLD disorders, thereafter the diagnostic criteria of Rascovsky et al. (2011) for bvFTD and of Gorno-Tempini et al. (2011) for svPPA and nfvPPA. Patients primarily diagnosed according to Neary et al. (1998) were re-classified using the Rascovsky et al. and Gorno-Tempini et al. criteria. Sixty-one out of 77 admitted patients with probable bvFTD and their caregivers gave their informed consent for inclusion in the FTLA registry study. The patients exhibited behavioural disinhibition, apathy, loss of empathy, and stereotypical or ritualistic behaviour. CERAD-Plus battery revealed neurocognitive deficits corresponding to the neuropsychological diagnostic criteria described by Rascovsky et al. (2011). Frontotemporal atrophy was found on MRI. In three out of 61 included patients the primary diagnosis was later revised (Dementia with Lewy bodies, AD, mixed dementia). Therefore, these patients were excluded from this study.

Nine of ten referred patients with progressive confrontation naming, single-word comprehension and object recognition difficulties and six of eight referred patients with non-fluent agrammatic speech impairment fulfilling the diagnostic criteria of svPPA and nfvPPA, respectively (Gorno-Tempini et al. 2011), and their carers agreed to be included in the FTLA registry. MRI of the svPPA patients showed left lateralised atrophy of the anterior temporal pole and the anterior fusiform gyrus (Landin-Romero et al. 2016). MRI of the nfvPPA patients revealed atrophy of the insular cortex and the middle and superior temporal, precentral and frontal gyri (Bisenius et al. 2016).

Baseline examinations comprised medical and family history, neurological and psychiatric symptoms and signs, routine blood laboratory and chemistry including serum vitamin B12, folate, T3, T4, TSH, HIV serology and TPHA, body weight as well as assessment of educational, occupational and social parameters. Moreover, as described below, neuropsychological tests were performed and neuropsychiatric and behavioural changes, activities of daily living and caregiver burden assessed at baseline and at follow-up (FU) visits 6, 12, 18 and 24 months after baseline (FU6, 12, 18 and 24) unless patients or carers dropped out earlier because of withdrawal of consent, terminal disease, need for professional care at home or admission to a residential home. MRI (standard protocol including T1, T2, FLAIR, MPRage) was repeated and clinical diagnoses were re-evaluated at the FU visits according to the established diagnostic criteria (Rascovsky et al. 2011; Gorno-Tempini et al. 2011).

From 2015 patients also underwent FDG-PET and CSF routine examinations including neurodegenerative parameters (Tau, phopho-tau, Aß-1-42).

### Scales

#### Caregiver burden

Caregiver burden was assessed using the Caregiver Strain Index (CSI) (Robinson 1983) from the onset of the registry (2007). After 2009, the Zarit Caregiver Burden Interview (ZBI) (Zarit et al. 1980) was added to the protocol for better comparability of the present study with a multicentric Austrian registry on Alzheimer’s disease (PRODEM; www.alzheimer-gesellschaft.at).

The CSI (Robinson 1983) consists of 13 dichotomic questions addressed to the caregiver about the presence/absence (scores 1/0) of restrictions in work, leisure, family life and personal plans, impairment of sleep, emotional changes, shocking experiences with the patient, financial burden, exhaustion, and excessive demands. A CSI total score of ≥ 7 indicates major caregiver strain.

The ZBI (Zarit et al. 1980) is also a self-rating questionnaire for caregivers comprising 22 questions about felt and experienced restrictions in privacy, personal contacts, social life and relationships, financial burden, excessive demands of the client and health problems. The ZBI also asks for feelings of insufficiency and guilt, negative emotions, thoughts about putting an end to caregiving and overall caregiver burden. The scores of the single domains range from zero to four (0 = never, 4 = nearly always), the total scores from zero (no) to 88 (maximum caregiver burden). ZBI total scores between 0 and 20 indicate no or little, 21–40, mild to moderate, 41–60, moderate to severe, and > 60, severe caregiver burden. A total score of 26 and higher indicates a significant risk for exhaustion and mental health problems (Schreiner et al., 2006).

CSI and ZBI address similar subject areas with few exceptions. The CSI asks for strain (according to the Oxford dictionary pressure, because of great demands) and the ZBI for burdens related to caregiving (Oxford dictionary: duties, responsibilities that cause difficulties). The ZBI is more detailed than the CSI. We hypothesized that, although there are differences between the two inventories, CSI and ZBI might lead to comparable results. The use of both inventories, however, might corroborate the results of the study.

#### Neuropsychological tests

The CERAD-Plus battery (Schmid et al. 2014) was applied for a global cognitive evaluation at baseline. It comprises subtests of letter and category fluency, naming, learning and immediate and delayed recall of words, copying and drawing from memory of geometric figures, the trail making tests A and B and the Mini-Mental State Examination (MMSE) (Folstein et al. 1975). The MMSE was taken as an indicative measure for global cognitive performance and repeated at FU visits. Frontal lobe functions were tested using the Frontal Assessment Battery (FAB) (Dubois et al. 2000), which consists of five neuropsychological subtests (conceptualization-umbrella term, mental flexibility/phonemic fluency-S-words, motor programming-Luria test, sensitivity to interference-conflicting instructions, inhibitory control-Go–No-Go paradigm), and one test of prehension behaviour (grasp reflex). Ratings of the single subtests range from 0, severe impairment, to 3, no impairment. Maximum possible sum score is 18 (maximum performance).

#### Frontal Behavioural Inventory (FBI)

Caregivers rated changes of behaviour and personality of the patients using this questionnaire for semiquantitative assessment of severity and frequency of 24 behavioural domains (Score 0 = none, 1 = mild, occasional, 2 = moderate, 3 = severe, present most of the time; Kertesz et al. 1997). The sum scores range from 0 (no impairment) to 72 (maximum behavioural impairments). A score of 28 or higher suggests significant behavioural deficits.

#### Neuropsychiatric Inventory (NPI)

Frequency (range 1–4) and severity (1–3 points) of ten categories of neuropsychiatric symptoms related to dementia and two somatic functions (impaired sleep and eating behaviour) were rated. Frequency and severity scores of all categories were multiplied and then summarized. Sum scores range from 0, symptomless, to 144, maximum impairment (Cummings et al. 1997). The scores of the single categories were added (“clustered”) according to Garre-Olmo et al. (2010) (Psychotic cluster: delusions, hallucinations; emotional cluster: agitation/aggression, depression/dysphoria, anxiety, irritability; behavioural cluster: elation/euphoria, apathy, disinhibition, aberrant motor behaviour; somatic cluster: sleep and eating behaviour).

#### Lawton Instrumental Activities of Daily Living (IADL) Scale

Caregivers rated the performance of the patient in eight domains of IADL. Sum scores range from 0, low function, dependence, to 16, high function, independence (Lawton and Brody, 1969).

#### The Barthel Index (BI):

The study team asked the caregivers for ten basic functions of activities of daily living (ADL). Sum score range between 0 and 100. The higher the score, the less dependent is a client (Mahoney and Barthel 1965).

### Statistical methods

All data were tabulated descriptively using appropriate summary statistics. Between-group differences in categorical variables were compared using the *χ*^2^ test. Assumptions of normal distribution for continuous variables were tested with the Kolmogorov–Smirnov test with Lilliefors correction. Normally distributed continuous variables were compared using analysis of variance or Welch’s ANOVA in case of variance heterogeneity. The Kruskal–Wallis rank analysis of variance was applied to compare parameters of the three patient groups in case of non-normally distributed continuous variables or ordinal variables and the Mann–Whitney *U* test to compare two groups. Adjustment for multiple testing was performed using the Bonferonni correction. For comparison of paired data from baseline to FU at 6, 12, 18 and 24 months, the Friedman rank analysis of variance was used.

To test for a potential univariate correlation between variables, Spearman’s rank correlation coefficient or a rank biserial correlation coefficient (in case that one of the variables was dichotomic) was used, respectively. Based on selected univariate statistically relevant variables multivariate risk models using general estimation equations (GEE) techniques were estimated. GEE techniques were used to account for the repeated measurements over time. Furthermore, for the individual measurements at baseline and after 12 and 24 months the most influential factors in terms of highest standardized beta using a linear regression model were presented. A two-sided *p* value < 0.05 was taken as uncorrected statistical significance level; therefore, all inferential results are descriptive. For statistical analysis the statistical computing software R Version 3.6.1 (R Foundation for Statistical Computing, Vienna, Austria; URL http://www.R-project.org) was used.

## Results

### Patient’s data and results at baseline

In 46 of 58 bvFTD, 9 of 10 svPPA and 6 of 8 nfvPPA patients of the FTLA registry the data set was complete. Demographic, educational, clinical data (neuropsychological and neuropsychiatric findings and ADL) and family status at baseline are summarized in Table 1. Age, male–female ratios, years of formal education and the percentage of patients living with their spouses or partners were similar in the three groups (*p* > 0.05). Disease duration (history of behavioural, cognitive or dysphasic symptoms) tended to be longer in bvFTD than in svPPA and nfvPPA (*p* = 0.06 and 0.09, respectively).

**Table 1:** Baseline patient characteristics of the FTLD patients

	bvFTD	svPPA	nfvPPA	Total
	*N* = 46	*N* = 9	*N* = 6	*N* = 61
	75%	15%	10%	100%
Age (years)				
Mean	70	69.7	75.1	69.7
SD	9.2	11.3	8.6	9.5
Median	70	69	76	70
IQR	14	13.8	7.8	14.3
Female				
*N*	28	3	3	34
%	61%	33%	50%	56%
Formal education (years)				
Mean	10.8	10.8	10.4	10.7
SD	2.9	2,1	2,5	2,7
Median	11	11	11	11
IQR	3.3	2.5	3	3.3
Disease duration (months)				
Mean	36.6	24.6	13	32.4
SD	34.8	19.4	11.2	31
Median	24	20	8.5	24
IQR	43	33	15.3	27.5
Married, partnership				
Yes				
*N*	27	8	4	39
%	58.70%	88.90%	66.60%	63.90%
CERAD-PLUS sum scores				
Mean	49.6	35	45.8	47.2
SD	18.8	18.5	20.1	19.3
Median	49	26	34	48
IQR	27.5	24	31	30
MMSE sum scores*				
Mean	22.2	15.1	18	20.9
SD	5.2	8.7	8.8	6.4
Median	24	16	17	22
IQR	8.3	15	16	10
FAB sum scores				
Mean	11.6	11.6	12	11.6
SD	4.5	3.7	4.3	4.3
Median	12	12	13	12
IQR	6	5	8	6
FBI sum scores*				
Mean	26.7	14.4	13.8	24.7
SD	14.6	8.1	10.5	14.7
Median	26	12.5	11	24
IQR	21.5	10.5	5	21.5
NPI sum scores*				
Mean	31	14.5	9.5	27.6
SD	26.7	13.7	8.2	26.1
Median	23	9	8	20
IQR	32.5	18.5	10.3	28.8
Barthel Index sum scores				
Mean	84.6	98.8	93.1	88.3
SD	26.9	3.5	13.7	23.4
Median	95	100	97.5	100
IQR	15	0	5	10
IADL sum scores (%)*				
Mean	53.5	79.6	79.6	58.9
SD	33.8	20.1	35	33.6
Median	56	88	100	63
IQR	69	40	21	63

*bvFTD* behavioral variant of frontotemporal degeneration, *FAB* Frontal Assessment Battery, *FBI* Frontal Behavioral Inventory, *FTLD* frontotemporal lobar degeneration, *IADL* Instrumental activities of daily living, *IQR* interquartile range, *MMSE* Mini-Mental State Examination, *NPI* Neuropsychiatric Inventory, *nfvPPA* non-fluent variant primary progressive aphasia, *SD* standard deviation, *svPPA* semantic variant primary progressive aphasia

*Comparison using Kruskal–Wallis test revealed *p* value < 0.05, see text

### Caregivers

The baseline data of the caregivers are summarized in Table 2: age and female/male ratios were similar in the three groups (*p* > 0.05). Spouses and partners were the largest group of caregivers. There were no statistically significant differences between the three cohorts in the proportion of spouses/partners, children or other relatives among caregivers (*p* > 0.05).

**Table 2:** Baseline characteristics of caregivers

	bvFTD *N* = 46 75%		svPPA *N* = 9 15%	nfvPPA *N* = 6 10%		Total *N* = 61 100%
Relationship to patient						
Spouse/partner						
*N*	27		7	4		39
%	58%		77.80%	67%		65%
Child						
*N*	12		1	1		13
%	26%		11.10%	16.50%		21%
Other						
*N*	3		1	1		5
%	7%		11.10%	16.50%		8%
Missing						
*N*	4		0	0		4
%	9%		0%	0%		6%
Age (years)						
Mean	57.4		66.2	59.5		59
SD	11.1		7.8	28		12.8
Median	59		64	62.5		61.5
IQR	18		12	40		17.8
Female						
*N*	26		8	3		36
%	57%		88.90%	50%		59%
Zarit Caregiver Burden Scale (ZCBI sum scores)						
Mean	26.9		17	12.8		25.5
SD	19.9		9.4	9.8		19.6
Median	25		16	16		20.5
IQR	30.8		16	16		29.8
Caregiver Strain Index* (CSI sum scores)						
Mean	5.5		2.9	1.6		5
SD	3.4		2.3	2.1		3.4
Median	5		3	1		5
IQR	6		3.5	2		6

*bvFTD* behavioural variant of frontotemporal degeneration, *CSI* Caregiver Strain Index, *IQR* interquartile range *Other* daughter-in-law, son-in-law, sister and brother, *nfvPPA* non-fluent variant primary progressive aphasia, *SD* standard deviation, *svPPA* semantic variant primary progressive aphasia, *ZBI* Zarit Caregiver Burden Interview

*Comparison using Kruskal–Wallis test revealed *p* value < 0.05, see text

### Neuropsychological measures (CERAD-Plus, MMSE, FAB), neuropsychiatric symptoms (NPI, FBI) and dependency (IADL, BI) at baseline and during FU

In the bvFTD group, mild to moderate impairment was found in the MMSE at baseline. MMSE sum scores were significantly higher in bvFTD than in svPPA (*p* = 0.05), and similar in svPPA and nfvPPA as well as in bvFTD and nfvPPA (*p* > 0.05) (Table 1). The CERAD-Plus sum scores were not significantly different between the three groups (*p* > 0.05). The FAB revealed comparable moderate impairments of frontal lobe functions in all three groups (Table 1).

Significant neuropsychiatric and behavioural impairments were found at baseline. FBI sum scores reflected more severe behavioural impairments in bvFTD than in svPPA (*p* = 0.05) and nfvPPA (*p* = 0.02). The baseline NPI sum scores in bvFTD patients were higher than in nfvPPA patients (*p* = 0.02). Basic activities of daily living (BI) tended to be more affected in bvFTD than in svPPA patients (*p* = 0.08), and were similar in bvFTD and nfvPPA. IADL were more impaired in bvFTD than in svPPA (*p* = 0.01) and in nfvPPA (*p* = 0.05).

The number of study participants decreased during FU: 45 patients (32 bvFTD, 8 svPPA and 5 nfvPPA patients) were seen at FU6, 37 (25 bvFTD, 7 svPPA and 5 nfvPPA patients), at FU12, 25 (15 bvFTD, 7 svPPA and 3 nfvPPA patients), at FU18, and 20 patients (10 bvFTD, 7 svPPA and 3 nfvPPA patients), 2 years after BL (FU24). Reasons for drop-out were inability to track the patient, intercurrent diseases, need for professional care at home, admission to a nursing home, withdrawal of consent, or death. Four patients died during the study. Death occurred at home or in a nursing home.

**Fig. 1:**
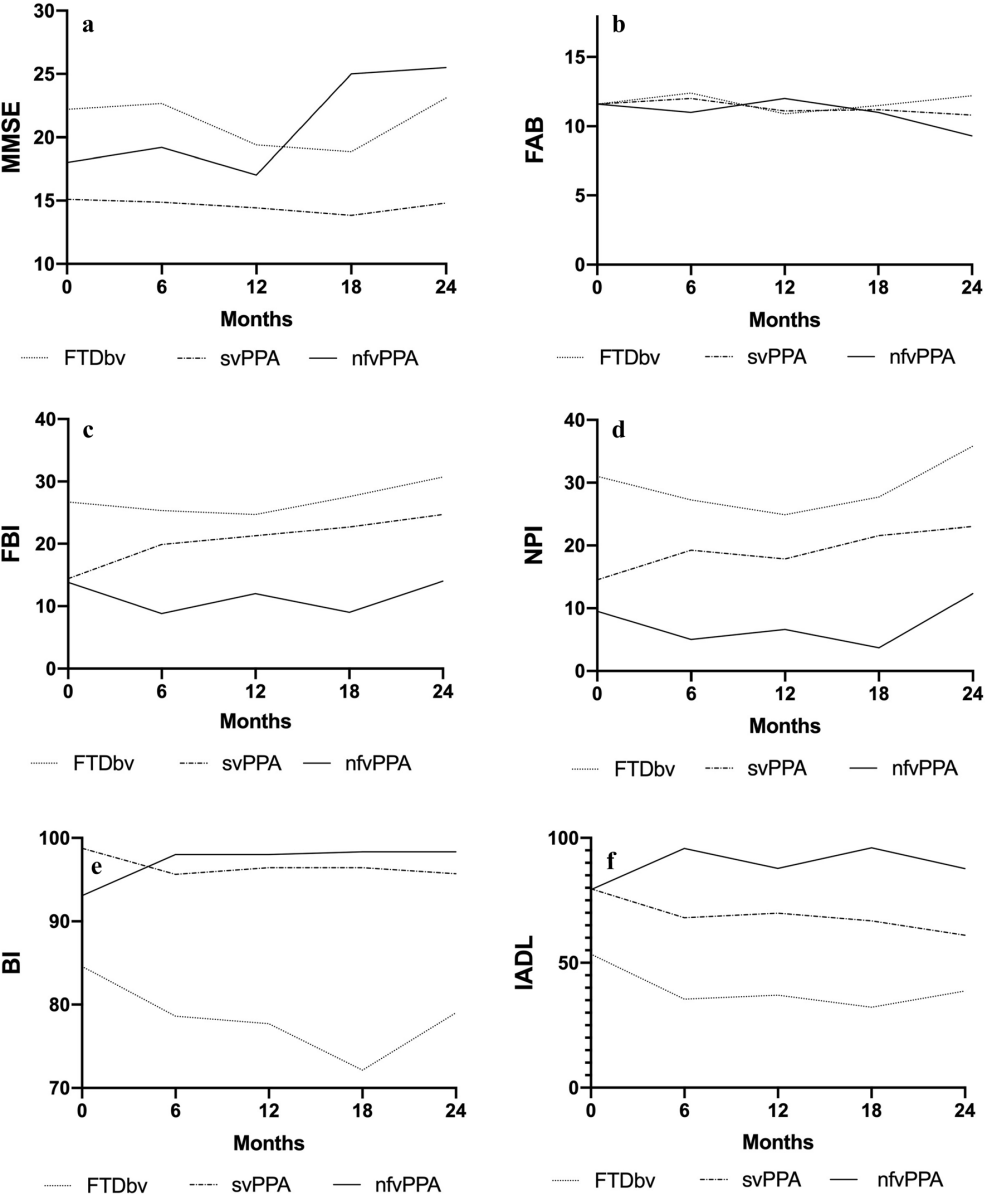
Mini-Mental State Examination mean sum scores (**a**), Frontal Assessment Battery mean sum scores (**b**), Frontal Behavioural Inventory mean sum scores (**c**), Neuropsychiatric Inventory mean sum scores (**d**), Barthel Index mean sum scores (**e**) and Instrumental activities of daily living mean sum scores (**f**) at baseline and follow-up visits. *BI *Barthel Index, *bvFTD* behavioural variant of frontotemporal degeneration, *FAB* Frontal Assessment Battery, *FBI* Frontal Behavioural Inventory, *IADL *Instrumental activities of daily living, *MMSE* Mini-Mental State Examination, *nfvPPA* non-fluent variant primary progressive aphasia, *NPI* Neuropsychiatric Inventory, *svPPA* semantic variant primary progressive aphasia

In bvFTD and svPPA, the MMSE sum scores did not significantly change during FU (*p* > 0.05; Fig. 1a). However, patients dropping out during FU had lower MMSE sum scores than those remaining in the study: patients lost to FU at FU6 had baseline MMSE sum scores of mean 20, median 18, compared to patients still seen at FU6: baseline MMSE 23.2 and 25, respectively. Patients lost between FU6 and FU12 had a MMSE sum score at FU6 of mean 21.5, median 24, those seen at FU12: 23.3 and 25, respectively. In the nfvPPA group, higher mean MMSE sum scores were found at FU18 and FU24 than at baseline, FU6 and FU12. However, only five patients returned to FU6 and 12, and only three patients to FU18 and 24. Therefore, the late FU data of this group is not representative and needs to be re-assessed in future studies.

The FAB sum scores did not significantly change over time (Fig. 1b). However, patients with comparatively worse FAB sum scores tended to drop out during FU (e.g., FAB sum scores at baseline of patients also seen at FU6: mean 13.3, median 15, patients lost to follow-up between baseline and FU6: 8 and 7, respectively. FAB sum scores at FU6 of patients also seen at FU12: 12.5 and 13, patients lost between FU6 and FU12: 11.4 and 11.5, respectively).

Statistically significant deterioration of the FBI sum scores was seen during FU in svPPA (*p* = 0.01) and not in bvFTD and nfvPPA patients (Fig. 1c). Moreover, significant deterioration of NPI sum scores was seen in svPPA patients (*p* = 0.02) (Fig. 1d). The most frequent and severe neuropsychiatric symptoms were apathy (median frequency score 3, median severity score 2, median caregiver distress score 1) followed by appetite changes (3, 1, 1), agitation (2, 1, 1), anxiety (2, 1, 2) and irritability (1.5, 1, 1.5, respectively).

BI was stable in svPPA and nfvPPA during FU until the end of the study, and deteriorated until FU18 in bvFTD (*p* = 0.03) (Fig. 1e). The IADL sum scores deteriorated significantly over time in the bvFTD group only (*p* = 0.04) (Fig. 1f).

### CSI and ZBI sum scores at baseline and during follow-up

**Fig. 2:**
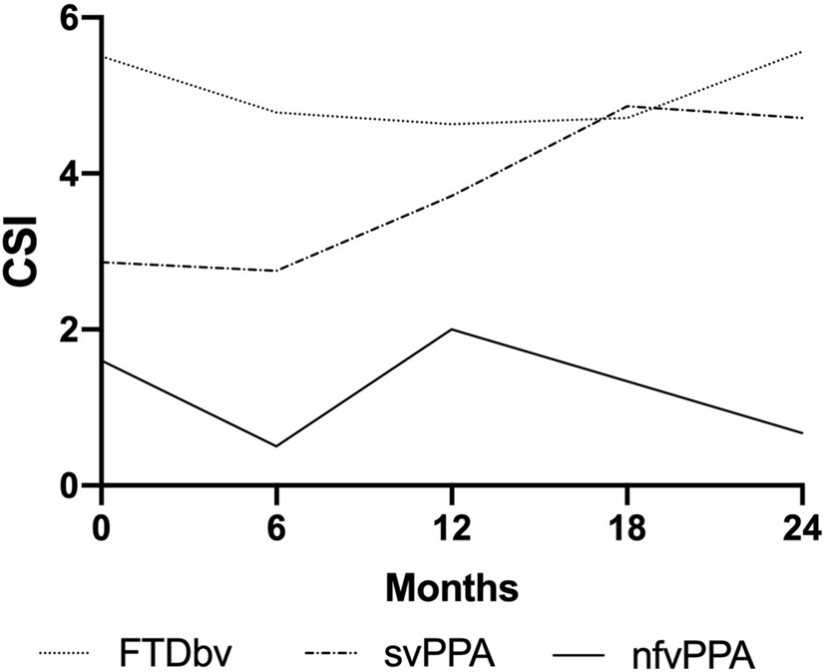
Caregiver Strain Index (CSI) mean sum scores at baseline and follow-up visits. Abbreviations Fig. 2 *bvFTD* behavioural variant of frontotemporal degeneration, *nfvPPA* non-fluent variant primary progressive aphasia, *svPPA* semantic variant primary progressive aphasia

CSI sum score at baseline was highest in bvFTD, but did not exceed the cutoff of 7 described in the literature as substantial caregiver strain (Robinson 1983) (Table 2). CSI sum scores were intermediate in svPPA, and lowest in nfvPPA. Statistical analysis revealed significantly higher strain (CSI scores) in bvFTD than in nfvPPA (*p* = 0.02), but CSI sum scores were not statistically significantly different in bvFTD and svPPA, and in svPPa and nfvPPA (*p* > 0.05). In the svPPA group, CSI sum scores deteriorated significantly between baseline and FU24 (p = 0.03), while it remained stable in the bvFTD and nfvPPA patients (Table 2, Fig. 2).

*BI* Barthel Index, *FAB* Frontal Assessment Battery, *FBI* Frontal Behavioural Inventory, *IADL* Instrumental activities of daily living, *MMSE* Mini-Mental State Examination, *NPI* Neuropsychiatric Inventory.

**Fig. 3:**
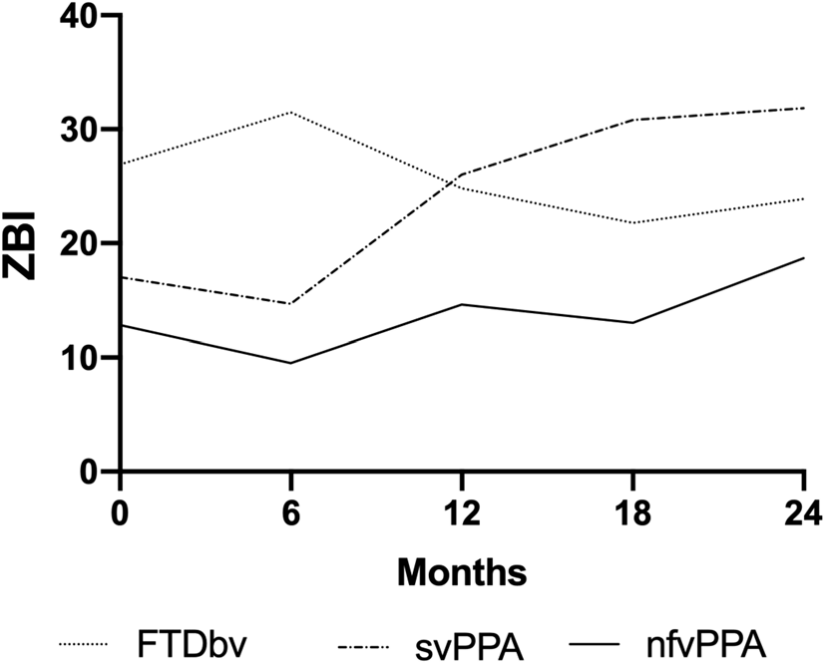
Zarit Burden Interview (ZBI) mean sum scores at baseline and follow-up visits. Abbreviations see Fig. 3

ZBI sum scores were assessed only in 37 patients at baseline, 33 at FU6, 28 at FU12, 19 at FU18 and 20 patients at FU24 (see also Patients and Methods). ZBI baseline sum scores revealed mild to moderate caregiver burden in bvFTD (mean 26.9; median 25). These scores corresponded to the cutoff sum score of 26 suggesting a significant risk of caregiver burden for mental health problems (Schreiner et al. 2006). In svPPA and nfvPPA patients the ZBI mean and median scores were somewhat lower than in bvFTD, the difference was not statistically significant (Table 2). During FU, the ZBI sum scores of caregivers of the bvFTD and nfvPPA patients did not significantly change, but increased significantly in caregivers of svPPA patients (*p* = 0.04) (Fig. 3).

*BI* Barthel Index, *bvFTD* behavioural variant of frontotemporal degeneration, *FAB* Frontal Assessment Battery, *FBI* Frontal Behavioural Inventory, *IADL* Instrumental activities of daily living, *MMSE* Mini-Mental State Examination, *NPI* Neuropsychiatric Inventory, *nfvPPA* non-fluent variant primary progressive aphasia, *svPPA* semantic variant primary progressive aphasia

### Influence of various indices on caregiver burden

Using Spearman’s rank correlation baseline CSI sum scores correlated significantly with the ZBI sum scores (correlation coefficient: 0.813, *p* < 0.001).

CSI and ZBI sum scores correlated significantly with severity of behavioural impairment (FBI sum scores; correlation coefficients: 0.779 and 0.721, respectively, *p* < 0.001; NPI sum scores; correlation coefficient: 0.695 and 0.677, respectively, *p* < 0.001) and impairments of IADL (IADL sum scores; correlation coefficient: − 0.505 and − 0.393; *p* < 0.001 and *p* = 0.02, respectively). CSI and ZBI sum scores correlated highly significantly with the emotional and the behavioural clusters of the NPI (*p* < 0.0001 and < 0.0001, respectively). Moreover, CSI and ZBI sum scores correlated significantly with the somatic cluster (*p* = 0.004, *p* = 0.008).

Baseline CSI sum scores (but not ZBI sum scores) correlated with disease duration (correlation coefficient: 0.297, *p* = 0.04) and impairments of basic ADL (BI; correlation coefficient: − 0.37, *p* = 0.006). Correlations between ZBI and CSI sum scores and age and sex of the patient, years of formal education and MMSE and FAB sum scores were not significant (*p* > 0.05). Age and sex of the caregiver and relationship to the patient did not significantly correlate with CSI or ZBI sum scores (*p* > 0.05, Table 2).

GEE techniques identified risk factors for caregiver burden over time (BL, FU6, FU12, FU18 and FU24). Risk factors for high CSI and ZBI sum scores were high FBI (*p* = 0.007, *p* = 0.004) and low IADL sum scores (*p* = 0.009, *p* = 0.013) as well as the age of the caregiver (*p* = 0.005, *p* = 0.028). NPI was an influential risk factor for a high CSI, but not a high ZBI sum score (*p* = 0.001).

Accordingly, the multivariate linear regression model found the most influential risk factors for high CSI and ZBI sum scores to be the FBI sum score at baseline (*p* < 0.001, *p* < 0.001) and at FU12 (*p* < 0.001, *p* = 0.013, respectively). Moreover, higher caregiver age was a risk factor for a high CSI sum score (*p* = 0.009) at FU12. Worse IADL and NPI scores and higher caregiver age were risk factors for a high CSI (CSI; *p* < 0.001, *p* < 0.001 and *p* = 0.024, respectively) at FU24. At FU24, the most relevant risk factor for a high ZBI sum score was a lower IADL score (*p* < 0.001).

In summary, both the GEE technique and the multivariate linear regression revealed significant correlations between caregiver burden and severity of neuropsychiatric symptoms, particularly frontal behavioural abnormalities, impairments in IADLs, and high age of the caregiver.

## Discussion

To the best of our knowledge, this is the first study comparing caregiver burden in bvFTD, svPPA and nfvPPA from the time of diagnosis over a longer period of time (up to 24 months). In this observational study, we prospectively examined patient and caregiver related parameters hypothesized to underlie caregiver burden. The CSI was completed by all included caregivers, the ZBI only by 37 caregivers for reasons reported in the Patients and Methods section. CSI and ZBI differ to a minor extent regarding domains of caregiver burden, wording, and scoring. However, most items of the two scales overlap, and the sum scores of the ZBI and the CSI correlate highly significantly (*p* < 0.001). We, therefore, found similar results including similar correlations with neuropsychological, neuropsychiatric, behavioural and activities of daily living parameters.

Our study has limitations. First, the sample size was relatively small, especially for the svPPA and nfvPPA sub-groups, due to the rarity of these diseases, but comparable to other studies in this field (Koyama et al. 2018; Hsieh et al. 2016; Mioshi et al. 2013). The number of included patients and caregivers decreased during FU. A high drop-out rate of patients and caregivers during FU was also observed by others (Ransmayr et al. 2018; Hsieh et al. 2016; Boutoleau-Bretonnière et al. 2012; Chow et al. 2012) and is explainable by rapid clinical deterioration, withdrawal of consent, admission to a nursing home or death.

Second, we did not evaluate caregiver burden of family caregivers after care had been taken over by professional caregivers at home or the patient had been admitted to a nursing home. We also did not evaluate if burden and strain persisted after the death of a patient.

Third, our patients fulfilled the most recent established clinical diagnostic criteria. The clinical diagnoses were not neuropathologically verified and genetic testing was not performed.

Fourth, there was no progression of the MMSE and the FAB scores in our study during FU. Dropped-out patients are usually more seriously ill than those remaining in a FU protocol. In the present study patients who dropped out had lower MMSE and FAB scores than those remaining in the study. Therefore, in real life, neuropsychological and neuropsychiatric deterioration and caregiver burden might progress faster than reflected by the caregiver burden scores during FU visits in our study.

Fifth, the caregivers were incompletely characterized. Daily and overall duration of care, the health status of the caregivers, the financial burden of care, medical, financial or psychosocial support, resilience, and socioeconomic parameters were not assessed.

Sixth, the scales used to assess activities of daily living might not be sufficiently reliable for older cognitively impaired persons (Sainsbury et al. 2005; Sikkes et al. 2009). However, since we included patients with various diagnoses and different age in the FTLA registry study we chose the BI and the IADL scales as a compromise.

Differences between the three cohorts were found in disease duration and family status at baseline (Table 1). This finding corresponds to other studies (Karnatz et al. 2019; Koyama et al. 2018). BvFTD patients were likely to be included at a more advanced disease stage than patients with nfvPPA and svPPA. A diagnostic delay of around 30 months is not unusual in bvFTD. Impairment of language seems to lead earlier to the diagnosis than behavioural abnormalities and frontal-lobe-type cognitive deterioration (Borroni et al. 2015; Reus et al. 2018; Leroy et al. 2021).

Behavioural impairment is a key feature of bvFTD. The longer history of symptoms in bvFTD might contribute to the worse behavioral and neuropsychiatric impairments (FBI and NPI) and the more impaired functions in the activities of daily living (BI and IADL) at baseline, as compared to the other patient groups. On the other hand, the neurocognitive status (MMSE) was best in bvFTD, but impairments of language have probably contributed to lower MMSE sum scores in svPPA and nfvPPA than in bvFTD, as the MMSE is mainly based on language-dependent tasks.

Caregiver burden correlated significantly with neuropsychiatric symptoms and behavioural abnormalities, but not with cognitive performance. This is the most probable reason why CSI and ZBI sum scores were higher in bvFTD than in svPPA and nfvPPA patients. In the bvFTD group, caregiver burden was similar compared to that reported in a recent study (mean ZBI sum score 28.7; Besser and Galvin 2019). In the svPPA cohort, ZBI sum score at baseline corresponded to the respective scores in other studies (Koyama et al. 2018; Hsieh et al. 2016). Caregivers of nfvPPA patients in our study reported a mean ZBI sum score of 12.8 at baseline, the lowest in our analysis. Mioshi et al. 2013 presented caregiver data of 20 nfvPPA patients with similar results. However, in our study the groups of patients with nfvPPA and svPPA, two rare diagnoses, were small and patients were lost to FU, so that the results need to be interpreted with great caution and verified in future studies.

Patients and caregivers were observed from the time of diagnosis for up to 2 years. Neuropsychiatric symptoms and behavioural abnormalities were moderate at baseline in svPPA. In contrast to bvFTD and nfvPPA, they increased significantly in svPPA over time. Prior studies have shown that impairment of ADL, younger age at disease onset, depression and disease severity, and older age, female sex, depression, anxiety and financial distress of caregivers correlate with caregiver burden (Karnatz et al. 2019; Besser and Galvin 2019; Liu et al. 2018; Koyama et al. 2018; Kaizik et al. 2017; Diehl-Schmid et al. 2013). Our results confirm these findings partially showing a significant correlation of caregiver burden with disease duration. Disease duration usually correlates with disease severity and duration of care. In the present study, caregiver burden did not correlate with years of formal education of the patients, severity of cognitive impairment, relationship between caregiver and patient, age and sex of the patient, and sex of the caregiver. Caregiver burden was found to correlate with the age of the caregiver in the GEE and the multivariate regression and not in the Spearman rank correlation. Our study population was probably too small to get clear results on this question. Recent publications demonstrated higher caregiver burden in male patients and female caregivers (Karnatz et al. 2019; Besser and Galvin 2019), which we could not confirm.

CB was higher in bvFTD than in svPPA and nfvPPA at baseline, remained stable in bvFTD and nfvPPA and progressed in svPPA during 2 years of follow-up. These findings confirm other studies demonstrating that CB may stabilize over time in bvFTD and nfvPPA, but progresses in svPPA (Riedijk et al. 2008; Hsieh et al. 2016; Liu et al. 2018; Karnatz et al. 2019; Besser and Galvin 2019). Our findings correspond to studies, that showed an increase of the perceived burden of care in svPPA patients during FU, whereas in bvFTD a high level of caregiver burden remained unchanged or stabilized during FU (Hsieh et al. 2016).

Caregivers of bvFTD patients experience more severe CB than caregivers of Alzheimer’s disease (AD) patients. On the other hand, caregiver burden in bvFTD seems to be comparable to that in patients with Lewy Body Dementia (LBD), progressive supranuclear palsy (PSP), corticobasal syndrome (CBS) and similar or higher than in Parkinson’s Disease (PD) (Ransmayr et al. 2018; Ransmayr 2020; Besser and Galvin 2019; Liu et al. 2018; Grün et al. 2016). Mean ZBI sum scores of caregivers of bvFTD patients in our study (Table 2) were higher compared to published ZBI scores of caregivers for AD patients. Values taken from the literature were as follows: AD, 19 and 12, respectively (Ransmayr et al. 2018; Liu et al. 2018); PSP, 28 (Besser and Galvin 2019), CBS, 25 (Besser and Galvin 2019), LBD, 23 (Liu et al. 2018), PD, 26 (Grün et al. 2016), and CSI mean sum score 3.17 in PD (Ransmayr 2020). However, comparability across these studies is limited due to variability in age, disease duration, neurocognitive status, behaviour, psychosocial factors and test instruments.

Our study intended to contribute to an improved knowledge of caregiver burden in persons caring for FTLD patients. FTLD spectrum diseases are rare, and medical and social institutions may be less familiar with these diseases Wynn and Carpenter 2020) compared to more frequent conditions, such as AD or PD. Caregiver burden and strain was found already in early phases of FTLD. The study demonstrates that apart from daily duration of care, a common decision criterion for public allowances for caregivers, other patient and also caregiver-related parameters markedly contribute to CB, which should be included in the assessment of CB and CS. Caregivers of FTLD patients require special attention to detect burden of care early and to provide adequate support.

## Data Availability

The datasets generated during and/or analysed during the current study are available from the corresponding author on reasonable request.
